# Characterization of Hypersensitivity to Iodinated Contrast Media: Insights from a Six-Year Cohort of 26,465 Procedures

**DOI:** 10.3390/healthcare13121407

**Published:** 2025-06-12

**Authors:** Kanokkarn Pinyopornpanish, Cheeratikarn Phithakham, Pakorn Prakaikietikul, Chanchanok Aramrat, Kanokporn Pinyopornpanish

**Affiliations:** 1 Department of Internal Medicine, Faculty of Medicine, Chiang Mai University, Chiang Mai 50200, Thailand; kanokkarn.pinyo@cmu.ac.th; 2 Department of Pharmacy, Faculty of Medicine, Chiang Mai University, Chiang Mai 50200, Thailand; cheeratikarn.p@cmu.ac.th; 3 Department of Radiology, Faculty of Medicine, Chiang Mai University, Chiang Mai 50200, Thailand; japakorn113@gmail.com; 4 Department of Family Medicine, Faculty of Medicine, Chiang Mai University, Chiang Mai 50200, Thailand; chanchanok.aram00@gmail.com; 5 Global Health and Chronic Conditions Research Group, Chiang Mai University, Chiang Mai 50200, Thailand

**Keywords:** iodinated contrast media, hypersensitivity, cross-reactivity, delayed hypersensitivity, contrast allergy

## Abstract

**Background/Objectives:** Hypersensitivity to iodinated contrast media (ICM) agents is a significant concern in clinical practice, potentially limiting their use in essential medical imaging studies and interventions. This cohort study reflects real-world clinical settings, with the aim of characterizing patients with a history of an ICM allergy and to analyze the potential cross-reactivity between different ICM agents. **Methods**: We conducted a retrospective review of patients with a documented history of an allergy to ICM. Data were collected from a six-year period, (2018 to 2023), and included a total of 26,465 procedures carried out with contrast. Data on patient demographics, reaction characteristics, culprit ICM agents, and outcomes of re-exposure were analyzed. **Results**: One hundred and eighty-three patients were identified as being allergic to at least one type of ICM. The majority of reactions were immediate (60.7%) versus delayed (39.3%). The most common culprit agent was Ioversol (3.84%) and related to the total time used. Among those who were ever exposed to more than one agent, the highest rate of recurrent hypersensitivity reactions was observed between Iohexol and Iodixanol (three out of six cases) and between Iopromide and Iopamidol (one out of two cases). No recurrent hypersensitivity reaction rate was observed between Iodixanol and Iopamidol (0 out of 12 cases). The highest proportion of severe allergies among those with allergic reactions was 3/15 (20%) for Iodixanol. **Conclusions**: Allergic reactions to ICM are uncommon and mostly non-severe. Although our findings do not confirm immunologic cross-reactivity, the occurrence of recurrent reactions to different ICMs in certain cases underscores the need for careful clinical judgment when selecting appropriate agents.

## 1. Introduction

Iodinated contrast media (ICM) are widely used in medical imaging and catheterization to enhance the visualization of internal organs and the diagnosis of various conditions. However, allergic reactions to ICM are a significant concern in patients, as these reactions can range from mild to severe. Severe reactions, including anaphylaxis, occur in approximately 0.04–0.28% of cases [1,2]. Fatal reactions after ICM administration are rare, with an incidence of one out of 170,000 cases. The overall incidence of hypersensitivity reactions is estimated to be 1–3%, with immediate reactions reported in 0.2–3% of cases and delayed reactions in 0.5–3% [3,4].

ICM agents are categorized into four structural types based on their molecular configuration, ionic monomeric, nonionic monomeric, ionic dimeric, and nonionic dimeric, as described in Table 1. These classifications reflect variations in chemical structure, osmolality, and side chain composition, all of which influence pharmacologic behavior and immunogenic potential. The transition from high-osmolar ionic agents to low-osmolar nonionic contrast media (LOCM) has significantly reduced the incidence of adverse reactions, primarily due to improved tolerability and lower osmolality. In current clinical practice, LOCM and nonionic agents are predominantly used due to their superior safety profile [5].

Hypersensitivity reactions to ICM can be classified as immediate or delayed. Immediate reactions may involve IgE-mediated or non-IgE-mediated mechanisms, such as direct mast cell degranulation, and are collectively referred to as allergic-like reactions. In contrast, delayed reactions are typically T cell mediated [8]. Dermatologic manifestations account for approximately 70% of adverse reactions, with urticaria being the most common, followed by localized skin reactions and pruritus [9]. Several risk factors for hypersensitivity to ICM have been identified, including an age over 50, a history of prior ICM reactions, and underlying allergic conditions such as asthma [10]. Despite the availability of alternative diagnostic methods, management of patients with ICM hypersensitivity is challenging, as many still require imaging with contrast.

Cross-reactivity among ICM agents remains a subject of debate, with some studies suggesting that structural similarities, particularly in side chains, may contribute to this phenomenon. Hypersensitivity reactions are therefore thought to be influenced by the molecular structure of the agents involved, such as the presence of N-(2,3-dihydroxypropyl)-carbamoyl or N-methyl-carbamoyl side chains, which are common among several nonionic monomers [11]. Recent studies in this area have approached the investigations in several ways. A study by Rosado Ingelmo et al. [12] reported a strong association between Iodixanol and Iohexol. Another study by Vega et al. [6] grouped ICM agents based on their chemical structure after devising a hypothesis related to their cross-reactivity. To explore these associations further, we conducted a retrospective analysis of cross-reactivity among various ICM agents, categorizing them into four distinct groups based on structural characteristics.

This study aims to evaluate hypersensitivity reactions to ICM, analyze patterns of possible cross-reactivity by examining clinical recurrent hypersensitivity reactions between different agents, and assess real-world outcomes following re-exposure to alternative ICMs. The findings are intended to provide insights for the optimization of patient safety and the informing of clinical decision-making in the management of ICM-related hypersensitivity.

## 2. Materials and Methods

This retrospective study was conducted at Maharaj Nakorn Chiang Mai Hospital, a tertiary hospital in Northern Thailand. Data from all patients who underwent procedures using iodinated contrast agents between 1 January 2018 and 31 December 2023 were collated, with a focus on those who developed hypersensitivity reactions. The exclusion criterion was the absence of documentation regarding which specific ICM agent was administered.

We collected data on baseline characteristics, including age, gender, underlying diseases, and Naranjo scores. The Naranjo scores are calculated based on a structured, questionnaire-based tool developed to estimate the probability of an adverse drug reaction. The questionnaire consists of 10 weighted questions assessing factors including temporal relationships, alternative causes, drug levels, dose–response relationships, and previous patient experiences. The total score categorizes the likelihood of an adverse drug reaction as definite (≥9), probable (5–8), possible (1–4), or doubtful (≤0) [13]. Data pertinent to any reactions were also collected, including characteristics of the reactions, reaction severity, creatinine levels, history of drug allergies, history of iodinated contrast allergy, timing related to re-exposure, premedication protocols, and contrast agent-specific allergies. Additional data on whether patients received alternative contrast agents after the first reaction were collected, identifying which agents were used and any subsequent reactions.

### 2.1. Types of Hypersensitivity Reactions

The diagnosis of hypersensitivity to ICM in this study was based on clinical documentation of signs and symptoms occurring after the administration of ICM. Diagnoses were made with reference to established criteria from the American College of Radiology (ACR). Reactions were classified as either immediate or delayed. Immediate reactions occurred within 6 h of exposure and included clinical presentations including urticaria and anaphylaxis. Delayed reactions were identified as those which occurred after 6 h and included clinical presentations such as maculopapular rash and severe cutaneous adverse reactions (SCARs). The severity of reactions was categorized as non-severe or severe based on clinical criteria. Non-severe reactions included a transient rash or itching without systemic involvement, and severe reactions encompassed life-threatening conditions such as anaphylaxis or SCARs. The identification of allergies was evaluated collaboratively by doctors and pharmacists.

Severe reactions were defined in accordance with the ACR Manual on Contrast Media as life-threatening events or those with the potential to cause permanent morbidity. These include laryngeal edema with stridor, bronchospasm with hypoxia, diffuse erythema with hypotension, or anaphylactic shock. For delayed reactions, severe cutaneous adverse reactions (SCARs)—such as Stevens–Johnson syndrome (SJS), toxic epidermal necrolysis (TEN), and drug reaction with eosinophilia and systemic symptoms (DRESS)—were also included in the definition [14].

### 2.2. Statistical Analysis

All statistical analyses were performed using STATA statistical software version 16. Descriptive statistics were used to summarize patient demographics and reaction characteristics, with continuous data presented as mean and standard deviation for normally distributed data and median and interquartile range for non-normally distributed data. Categorical data are presented as frequency and percentage.

## 3. Results

Out of a total of 26,465 procedures involving ICM, 21,272 were analyzed further. Procedures involving repeat use of the same ICM on the same patient were excluded to avoid duplication of reaction data. Out of the 21,272, allergic reactions were reported in 194 (0.91%) cases. Table 2 shows the details of allergies to ICM, indicating that Ioversol was the agent most commonly associated with an allergic reaction (3.84%).

One hundred and eighty-three patients were identified as being allergic to at least one contrast agent. Patient demographic and comorbidities are described in Table 3. The mean age was 55.14 years (SD 16.99, range 6–90), with females comprising 58.47% of the cohort. Common comorbidities included heart disease (25.68%), cancer (28.42%), diabetes mellitus (6.01%), and hypertension (6.01%). A history of drug allergies was reported in 27.32% of patients, while 7.65% had a history of contrast allergies. Skin reactions were the most frequently observed adverse events, affecting 96.17% of the patients with an allergic reaction. Most of these cases were non-severe (91.26%). The average Naranjo score was 4.85 (SD 1.56).

Table 4 shows patients who presented with allergic reactions following exposure to more than one agent. The highest rates of recurrent hypersensitive reactions were observed among patients who received both Iohexol and Iodixanol (three out of six cases), and those who received both Iopromide and Iopamidol (one out of two cases). No recurrent hypersensitivity reactions were observed among patients who received both Iodixanol and Iopamidol (0 out of 12 cases).

Following analysis of 16 severe cases, including anaphylaxis and SCARs, we investigated factors associated with severe reactions, as shown in Table 5. The proportion of severe allergies among those with allergic reactions was 8/77 (10.39%) for Iohexol, 3/32 (9.39%) for Iodixanol, 3/34 (8.82%) for Iopamidol, and 2/35 (5.71%) for Ioversol. Among these cases, the majority did not have any alternative contrast use reported. Of the three cases that underwent re-exposure, one case reported an allergic reaction to another agent (Iohexol → Iopamidol).

## 4. Discussion

This study revealed that Iohexol was the most frequently used initial iodinated contrast agent, while Ioversol accounted for the highest proportion of allergic reactions (3.84%). Allergic reactions were reported in nearly one percent of cases, with cutaneous reactions being the most frequent type of allergic response to ICM. Most frequently recurrent hypersensitivity reactions were observed as associated with Iohexol and Iodixanol in patients experiencing any allergic reaction.

Consistent with a previous study, Iohexol was the most frequently used agent in our institution, reflecting its established safety profile [15]. The use of nonionic, LOCM agents has been shown to be safer than ionic, high-osmolar agents in many aspects, including a lower risk of nephrotoxicity and adverse reaction [16,17,18,19,20]. However, other studies present varying findings. Zeng Wan et al. [21] reported Iomeprol and Iodixanol as the agents most frequently used, with differences probably due to contrast agent selection and availability at each hospital.

Our reported incidence of ICM-related allergic reactions is in alignment with the prior literature but there is a degree of variation in culprit agents. For instance, while Ioversol was most frequently implicated in our study, a finding also reported in a study from China [22], a Japanese study [23] found that Iomeprol had the highest reaction rate (3.9%), whereas Ioversol had the lowest (1.8%). In Korea, Iopromide, Iohexol, and Iopamidol were commonly associated with adverse reactions [24], although usage frequency data for each contrast agent was not provided in that study.

Cutaneous reactions, such as urticaria and maculopapular eruptions, were the most frequent clinical manifestations [25,26,27,28], with immediate reactions predominating in most reports. For example, Witchaya S. et al. [29] noted that 77.1% of reactions occurred within minutes of administration. However, the proportion of severe reactions has been shown to vary widely depending on classification criteria: Sohn K. et al. [25] reported severe reactions in 38.6% of cases, while others reported significantly lower rates ranging from 1.6% to 10% [30,31].

A key finding from our study was the rate of recurrent hypersensitivity reactions among patients re-exposed to group A agents, specifically between Iohexol and Iodixanol, which was as high as three out of six cases. The mechanisms underlying these recurrences are not fully understood but the hypothesis is that there are shared molecular structures, particularly the N-(2,3-dihydroxypropyl) and N-(2,3-dihydroxypropyl)-N-methyl-carbamoyl side chains. This warrants further investigation. Although contrast media molecules are small (typically less than 1 kDa), they may act as haptens, potentially triggering immune-mediated responses in sensitized individuals. Hypersensitivity reactions to ICM may involve multiple immunological pathways, with various molecular structures potentially triggering T cell-mediated [32,33], IgE-mediated, or non-IgE-mediated mechanisms [34,35].

Several studies support the structural hypothesis of cross-reactivity. Schmid et al. [36] reported a significantly higher skin test positivity rate within the same structural group (48.3%) compared to across groups (17.7%). Similarly, Sohn K. et al. [26] reported that among 482 skin tests performed in patients with immediate hypersensitivity reactions to LOCM, skin prick tests were positive in 3.1% of cases, while intradermal tests showed a significantly higher positivity rate of 51.8%. Cross-reactivity was observed in 21.5% of cases where the LOCM shared a common N-(2,3-dihydroxypropyl) carbamoyl side chain, compared to 13.3% among those without shared side chains. They also found that the overall recurrence rate of hypersensitivity upon re-exposure was 12.3% when a skin test-negative, non-culprit LOCM was used, a figure significantly lower than the 43.8% recurrence rate when the culprit agent was reused. Among cross-reactive pairs, iohexol and iomeprol showed the highest cross-reactivity rate at 36.3%. Dona et al. demonstrated that even with skin test-guided selection of alternative agents, 35.64% of patients still experienced allergic reactions to more than one culprit agent [37]. To mitigate these risks, guidelines such as those proposed by Francisco V. et al. [6] recommend the selection of alternative agents from structurally different groups when the culprit agent is identified. Our findings support this strategy, as agents such as Iopamidol (group D) demonstrated lower rates of recurrent hypersensitivity reactions, especially in cases of an allergy to group A.

The strength of our study lies in its relatively large sample size, enabling a detailed analysis of hypersensitivity incidence and re-exposure outcomes. By comparing culprit reactions with overall usage, the study provides a clearer estimate of reaction rates. Conducted in a real-world clinical setting, the findings are directly applicable to routine practice and reflect a diverse patient population with differing demographics and a wide range of health conditions. However, some limitations should be noted. The retrospective nature of the design limits the ability to establish cause-and-effect relationships and may introduce bias due to incomplete records. The absence of standardized allergy testing, such as skin testing or drug challenge procedures, represents a significant limitation, as it prevents definitive confirmation of immunological mechanisms. Without these confirmatory tests, the classification of reactions and the assessment of potential cross-reactivity rely on clinical judgment and observational data. The multidisciplinary input from pharmacists and clinicians did help in the refining of the attribution of causality and improved the potential for the transferability of the findings; however, the results should be interpreted with caution. Additionally, the small number of re-exposure cases and severe reactions limits statistical power, making it difficult to identify reliable predictors or outcome patterns within these subgroups. Finally, recurrent hypersensitivity reactions could only be evaluated among contrast agents that were used during the study, further limiting the generalizability of our findings.

## 5. Conclusions

This study offers insight into the patterns of hypersensitivity and possible cross-reactivity among iodinated contrast media. Most reactions were mild, but some severe and recurrent cases suggest the need for careful agent selection. Further studies are needed to better understand risk factors, the role of premedication, and the use of structurally different agents. Switching to a medium with a different structural group may help reduce the chance of recurrence when the underlying mechanism is unclear.

## Figures and Tables

**Table 1:** Structure, class and osmolality of each ICM [6,7].

Group	Structure	Name	Class	Osmolality (mOsm/kg)
A	Containing N-(2,3-dihydroxypropyl)-carbamoyl sidechains	Iohexol	Nonionic monomer	640
		Ioversol	Nonionic monomer	702
		Iodixanol	Nonionic dimer	290
		Iomeprol	Nonionic monomer	726
B	Containing N-(2,3-dihydroxypropyl)-N-methyl-carbamoyl sidechains	Iobitridol	Nonionic monomer	915
C	Containing N-(2,3-dihydroxypropyl)-carbamoyl and N-(2,3-dihydroxypropyl)-N-methyl-carbamoyl sidechains	Iopromide	Nonionic monomer	590
D	Not containing N-(2,3-dihydroxypropyl)-carbamoyl and N-(2,3-dihydroxypropyl)-N-methyl-carbamoyl sidechains	Iopamidol	Nonionic monomer	616

**Table 2:** Six-year report of total numbers of uses of iodinated contrast media and numbers of reactions.

Contrast Media	Reported Allergic Reactions	Total Number of Uses	Percentage of Allergic Reactions
Iohexol (A)	77	11,306	0.68
Iodixanol (A)	32	1958	1.63
Ioversol (A)	35	910	3.84
Iobitridol (B)	2	2617	0.08
Iopromide (C)	14	1215	1.15
Iopamidol (D)	34	3266	1.04
Total	194	21,272	0.91

The letters in parentheses indicate the group classification (A–D) of iodinated contrast media.

**Table 3:** Demographic data of patients who had an allergic reaction to at least one ICM.

Demographic Data	n = 183
Age (years), Mean ± SD	55.14 ± 16.99
Female gender, n (%)	107 (58.47)
Naranjo score, Mean ± SD	4.85 ± 1.56
Reactions, n (%)	
Skin	176 (96.17)
Respiratory system	19 (10.38)
Gastrointestinal system	4 (2.19)
Cardiovascular system	10 (5.52)
Neurological system	5 (2.73)
Severity, n (%)	
Non-severe	167 (91.26)
Severe	16 (8.74)
Immediate/delayed hypersensitivity, n (%)	
Immediate	111 (60.66)
Delayed	72 (39.34)
Underlying conditions, n (%)	
Asthma	6 (3.28)
Other respiratory disease	13 (7.10)
Urticaria	1 (0.55)
Diabetes mellitus	11 (6.01)
Dyslipidemia	7 (3.83)
Hypertension	11 (6.01)
Other cardiovascular disease	47 (25.68)
Cancer	52 (28.42)
History of drug allergy, n (%)	50 (27.32)
History of contrast allergy, n (%)	14 (7.65)
Creatinine (mg/dL), Mean ± SD	1.18 (0.39–7.23)
Premedication, n (%)	12 (6.56)

**Table 4:** Number of patients with recurrent hypersensitivity reactions upon re-exposure to ICM (n = 84 procedures).

	Iohexol	Iodixanol	Ioversol	Iobitridol	Iopromide	Iopamidol
Iohexol (A)	x	3/6	2/16	0/0	0/1	5/32
Iodixanol (A)	x	x	0/3	0/0	0/0	0/12
Ioversol (A)	x	x	x	0/0	0/1	3/11
Iobitridol (B)	x	x	x	x	0/0	0/0
Iopromide (C)	x	x	x	x	x	1/2
Iopamidol (D)	x	x	x	x	x	x

The letters in parentheses indicate the group classification (A–D) of iodinated contrast media. The number of reaction cases out of the total number of patients exposed to each specific pair of agents. An individual may have been exposed to the same agent more than once, but repeated exposures were not counted. ‘x’ indicates redundant comparisons or self-comparisons where data were not applicable.

**Table 5:** Details of severe cases.

	Characteristics	Underlying Conditions	Premedication	Reactions	Contrast That Led to Severe Reaction	Other Contrast Use
1	Male 23 Naranjo 4	Traumatic brain injury	No	Hypotension and wheezing	Iohexol 88	None
2	Female 60 Naranjo 8	Cancer	Yes *	Urticaria and wheezing	Iopamidol 100	None
3	Male 49 Naranjo 6	Mitral regurgitation	No	Urticaria, desaturation and hypotension	Iohexol 100	None
4	Male 68 Naranjo 3	Atypical chest pain, asthma	No	Faint, hypotension and dyspnea	Iohexol 100	None
5	Female 86 Naranjo 7	Heart disease Drug allergy	Yes *	Rash, angioedema and stridor	Iodixanol 100	None
6	Female 62 Naranjo 8	Heart disease, diabetes, hypertension, dyslipidemia	No	Urticaria, hypotension and desaturation	Ioversol 70	None
7	Male 45 Naranjo 4	Drug allergy	No	AGEP	Iodixanol 100	None
8	Female 67 Naranjo 7	Cancer, drug allergy	No	DRESS	Iohexol 90	None
9	Female 79 Naranjo 5	Respiratory, diabetes, hypertension	No	Hypotension and Respiratory failure	Iopamidol 100	Not allergic to Iodixanol
10	Male 73 Naranjo 4	Heart disease, drug allergy	No	Rash, wheezing, desaturation	Iohexol 90	None
11	Female 77 Naranjo 4	Hypertension, COPD, atrial fibrillation	No	Nausea, vomiting, wheezing, erythematous patch	Iodixanol 100	None
12	Female 69 Naranjo 3	Cancer	Yes *	Angioedema, dyspnea and desaturation	Iohexol 90	None
13	Male 63 Naranjo 7	Cancer	No	Itching, chest pain and dyspnea	Iohexol 90	Not allergic to Iopamidol
14	Male 49 Naranjo 8	Heart disease	Yes *	Hypotension	Ioversol 100	None
15	Female 64 Naranjo 2	Cancer with lung metastasis	No	Desaturation	Iohexol 90	Allergic to Iopamidol
16	Male 74 Naranjo 5	Asthma, diabetes	No	Bronchospasm	Iopamidol 100	None

AGEP = Acute Generalized Exanthematous Pustulosis; COPD = Chronic Obstructive Pulmonary Disease; DRESS = Drug Reaction with Eosinophilia and Systemic Symptoms; * Patients who received premedication were typically given oral prednisolone of 30 mg at 12 h and again at 2 h prior to contrast administration.

## Data Availability

The datasets used and/or analyzed during the current study are available from the corresponding author on reasonable request.

## Abbreviations

The following abbreviations are used in this manuscript:

ICM	Iodinated contrast media
SCARs	severe cutaneous adverse reactions
